# Malting and fermentation effects on antinutritional components and functional characteristics of sorghum flour

**DOI:** 10.1002/fsn3.525

**Published:** 2017-11-12

**Authors:** Pravin Ojha, Roshan Adhikari, Roman Karki, Achyut Mishra, Ujjwol Subedi, Tika Bahadur Karki

**Affiliations:** ^1^ Food Research Division Nepal Agricultural Research Council Lalitpur Nepal; ^2^ Department of Food Technology National College of Food Science and Technology Kathmandu Nepal; ^3^ Department of Biotechnology Kathmandu University Dhulikhel Nepal

**Keywords:** Fermentation, hydrogen cyanide, malting, sorghum

## Abstract

The research was aimed to observe the effect of malting and fermentation on antinutritional component and functional characteristics of sorghum flour. For whole sorghum flour, cleaned sorghum grain was milled to pass through 40 mesh sieve. For malting, cleaned sorghum grain was steeped in 0.2% calcium hydroxide solution for 24 hr and then germinated for 48 hr at 90% RH and 27 ± 2°C. Sprout was removed, dried in hot air oven at 50 ± 2°C for 24 hr and milled to pass through 40 mesh sieve. For fermented sorghum flour, 13.3 mg% diastase and 2 mg % pepsin (on the basis of whole sorghum flour weight) was added to cooked (88 ± 2°C) sorghum flour and left for 1 hr. *Lactobacillus plantarum* (10^7 ^cfu/g) was inoculated and incubated at temperature 30 ± 2°C for 48 hr. The fermented slurry was dried at 50 ± 2°C in hot air oven for 24 hr and milled to pass through 40 mesh sieve. The lower yield of sorghum flour was obtained compared to whole and malted sorghum flour. Germination of sorghum reduced phytate, tannin, and oxalate by 40%, 16.12% and 49.1%, respectively, whereas fermentation of sorghum flour reduced above by 77%, 96.7% and 67.85%, respectively. There was no significant change in hydrogen cyanide in malted sorghum flour compared to whole sorghum flour, but fermentation of sorghum flour reduced hydrogen cyanide by 52.3%. Bulk density and viscosity was significantly reduced by the malting and fermentation, whereas water absorption capacity and oil absorption capacity was markedly increased by the malting and fermentation. Fermented flour was good due to reduced ANF and improved functional property despite of lower yield.

## 1 INTRODUCTION

Sorghum is fifth important staple cereal and was considered to be drought resistant crop (Dlamini, Taylor, & Rooney, 2007; FAO 2006; Raihanatu, Modu, Falmata, Shettima, & Heman, 2011). It has been utilized as porridge, beer, unleavened bread, couscous composite blends, and ethnic beverages (Taylor, Schober, & Bean, 2006; Waniska, Rooney, & McDonough, 2004). Sorghum is a principle source of energy, protein, mineral including trace component like zinc and iron in diet for African and Indian population (FAO 2006; Mohammed, Mohammed, & Barbiker, 2011). Besides these nutrients, sorghum also contains high amount of phenolic acid, flavonoid, antioxidant, and condensed tannin (Awika & Rooney, 2004; Dykes & Rooney, 2007; Serna‐Saldivar & Rooney, 1995).

Sorghum is considered as food with low nutritional value (Raihanatu et al., 2011). Poor digestibility of sorghum and limited product diversification compared to other cereals limit the use of sorghum (Mella 2011).Further, sorghum contains anti‐nutritional factors like tannin, cyanogenicglucoside, phytic acid, trypsin inhibitor, and oxalate (Etuk, Okeudo, Esonu, & Udedibie, 2012; Mohammed et al., 2011). Due to these and other reasons, sorghum is categorized as of low nutritional value and a food for the poor. Low protein digestibility and mineral absorption are also associated with the presence of antinutritional factors (Mohammed et al., 2011; Scalbert et al., 1999). Various researches have revealed that the processing condition decreased antinutritional factors and increased the bioavailability of other nutrient in cereals and legumes (Adegunwa, Adebowale, & Solano, 2012; Mubarak, 2005; Osman, 2007; Yasmin, Zeb, Khalil, Paracha, & Khattak, 2008; Ogbonna 2012). Sorghum in Nepal is restricted to household purpose.

Application of flour in food application depends upon the functional property of flour (Igbabul, Bello, & Ani, 2014). Variation in functional properties of flour depends upon the source, their compositional structure, molecular confirmation, protein quality, its interaction with other food component (Chandra & Samsher, 2013). Replacement of wheat flour and preparation of composite flour in different food process application is related to functional properties of that flour (Chandra & Samsher, 2013; Noorfarahzilah, Lee, Sharifudin, MohdFadzelly, & Hasmadi, 2014).

The main objective of this research was to study the effect of traditional processing method with a few improvements on antinutritional component and functional properties of sorghum flour.

## 2 MATERIALS AND METHODS

### 2.1 Materials

White variety sorghum grains were purchased from the Chitwan, Nepal and packed in plastic bag. *Lactobacillus plantarum* was isolated in the laboratory of microbiology, National College, Kathmandu, Nepal. Amylase (1300 IU/g) and pepsin (1:10000) was purchased from central drug house Pvt. ltd., India.

### 2.2 Preparation of sorghum flour

Whole sorghum flour was prepared by milling cleaned sorghum flour in grinder (Ameet IS:4520, India) and passed through 40 mesh size screen (HT/Standard sieves). For malted sorghum flour, cleaned sorghum was steeped in 0.2% calcium hydroxide solution for 24 hr. The ratio of the solution and sorghum was 2:1. Water was drained and washed with distilled water and germinated in the RH chamber at 90% RH and 27 ± 2°C for 48 hr. Sprouts were manually removed and dried at 50 ± 2°C for 24 hr. Dried malted sorghum was milled in grinder (Ameet IS:4520, India) and passed through 40 mesh size screen (HT/Standard sieves) (Elkhalifa & Bernhardt, 2010; Ojha, Karki, & Maharjan, 2014). For fermented sorghum flour, whole sorghum flour mixed with water (1:2) was cooked for 10 min in 88 ± 2°C to gelatinize the starch. A total of 13.3 mg% diastase and 2 mg% pepsin was added at 30 ± 2°C and left for 1 hr. *Lactobacillus plantarum* (10^7 ^cfu/g) was inoculated and incubated at temperature 30 ± 2°C for 48 hr. The pH dropped from 6.2 to 4.3. Fermented slurry was dried at 50 ± 2°C for 24 hr. Dried fermented sorghum cake was milled in grinder (Ameet IS:4520, India) and passed through 40 mesh size screen (HT/Standard sieves) (Mukisa et al., 2012; Wedad et al., 2008).

### 2.3 Analysis

Milling yield refers to the percent of flour obtained from a given unit of kernels. It is also called as flour yield (House, 2006).

Flour yield=(weight of flour/initial weight of grain)×100%

Moisture content, Phytate, tannin, and oxalate of sorghum flour was determined as per described by Ranganna (2007). Hydrogen cyanide of sorghum flour was determined by AOAC (2005). For hydrogen cyanide, kjeldahl digestion flask was used to digest sorghum flour sample and distillate was collected in 250 ml volumetric flask containing NaOH (0.5 g in 20 ml) solution. It was first treated with 5% potassium iodide solution and titrated with 0.02 mol/L AgNO_3_ solution.

For bulk density, volume of 25 g of sample was measured using 50 ml graduated cylinder as adopted by Kanpairo, Usawakesmanee, Sirivongpaisal, and Siripongvutikorn (2012) with some modification. The bulk density was calculated as following relationship.

Bulk density=Weight of powder /Volume of powder

The method of Nwosu and Justina (2011) was adopted for determination of viscosity of 10% of flour using Ostwald viscometer. The method adopted by Sathe, Desphande, and Salunkhe (1982) was used for oil absorption. The density of oil (refined soybean oil) used was 0.92 g/ml. The flour sample and oil blend was prepared by magnetic stirrer and then centrifuged at 1233 g for 30 min. The oil absorbed was measured as percent absorbed by sorghum flour sample after decantation. Sorghum flour sample was mixed with water and then centrifuged at 402 g for 5 min and water absorbed was measured as percent absorbed by sorghum flour sample after discarding the supernatant (Nwosu and Justina 2011).

GenStat (Discovery Edition 12 developed by VSN International Limited) was used to perform analysis of variance (one way) to determine significant differences using the least significant difference (LSD) at 5% significance level. Triplicate data were used for computation.

## 3 RESULTS AND DISCUSSION

### 3.1 Yield of flour

**Figure 1:**
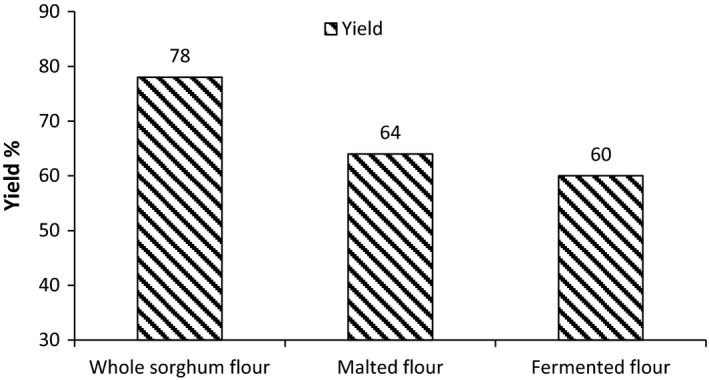
Effect of processing on yield of sorghum flour

The yield of the flour followed by the different processes applied is shown in Figure 1. The yield of the whole sorghum flour is found to be 78% and that of the malted flour is found to be 64% and fermented flour as 60%.

The low yield of flour may be due to removal of the bran and germ after sieving (Ackerson, Schemm, & Wagner, 1978). (Nwabueze and Atuonwu (2007) revealed 18.6% loss of dry matter in malted African breadfruit seed powder. Separation of sprout, respiration, and leaching loss might result in low yield of malted sorghum flour (Briggs, Hough, Steevans, & Young, 1981). The fermented flour has a least yield.

### 3.2 Antinutritional Components

#### 3.2.1 Phytic acid

**Figure 2:**
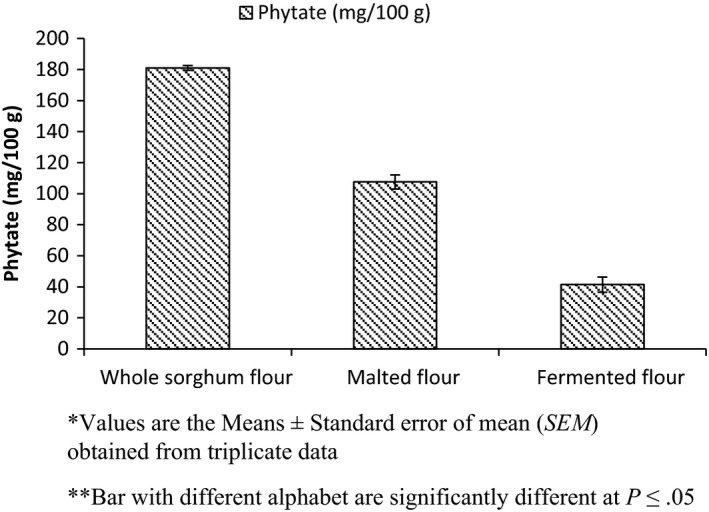
Effect of processing on phytic acid of sorghum flour

The effect of processing on phytic acid is shown in Figure 2. The result shows that malting and fermentation reduced phytate significantly up to 40% and 77%, respectively.

Mohammed et al. (2011) reported 317.65 mg phytic acid/100 g in sorghum flour and Rosa, Guerra‐Hernandez, and GarcõÂa‐Villanova (1999) reported 10.12 mg phytic acid/g in sorghum flour, which were greater than the result obtained, which might be due to varietal difference. Utilization of phytate as source of inorganic phosphate for germination, phytase activity, and leaching loss during soaking may result in reduction in phytate (Bau, Villaume, Nicolas, & Mejean, 1997; Beleia, Thu, & IDA, 1993; Valencia, Svanberg, Sandberg, & Ruales, 1999). The inherent phytase activity of sorghum during fermentation results in reduction in phytic acid (Reddy, Sathe, & Salunkhe, 1982). Lactic acid bacteria exhibit phytase activity during fermentation and may degrade phytate (Sreeramulu, Srinivasa, Nand, & Joseph, 1996). High reduction in phytate content of fermented sorghum flour compared to germinated sorghum flour may be due to low pH of fermented slurry and high activity of phytase in pH 4.5–5.0 (Valencia et al., 1999).

#### 3.2.2 Tannin

**Figure 3:**
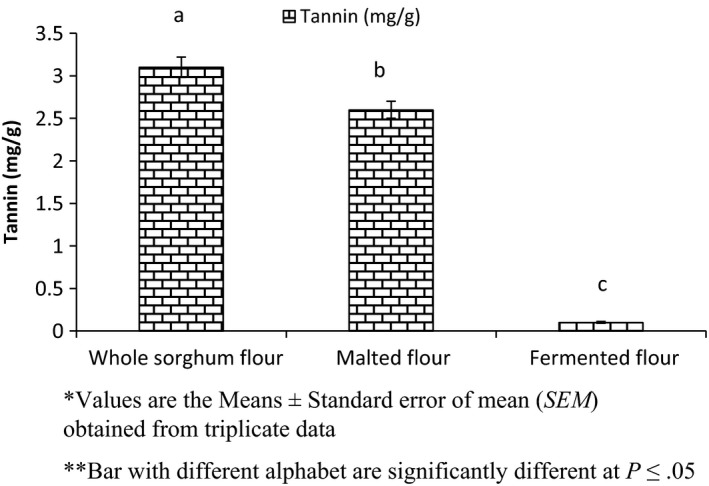
Effect of processing on tannin of sorghum flour

The effect of processing on tannin content of sorghum flour is shown in Figure 3. Malting and fermentation reduced the tannin content of sorghum flour to 2.6 mg/g and 0.1 mg/g, respectively, while unprocessed whole sorghum flour contain 3.1 mg/g.

These values were less than reported by Dlamini et al. (2007); Claver, Zhang, Li, Zhu, and Zhou (2010) but less than that of Mohammed et al. (2011). Shadad (1989) reported that light colored sorghum contains less tannin than dark colored tannin. Decreased tannin content during germination was observed in case of sorghum and millet (Chavan & Kadam, 1989). Ogbonna et al. (2012) revealed a decrease in tannin content due to leaching loss during steeping. Alkali treatment may also reduce tannin content (Babiker & el Tinay, 1993). Romo‐parada, Simard, & Larrea‐Reynoso, 1985 reported a 92% reduction in tannin content. Microbial activity may reduce the tannin content of fermented sorghum flour and tannin acyl hydrolases is responsible for the tannin reduction in fermented sorghum flour (Grewal, 1992; Schons, Battestin, & Macedo, 2012).

#### 3.2.3 Oxalate

**Figure 4:**
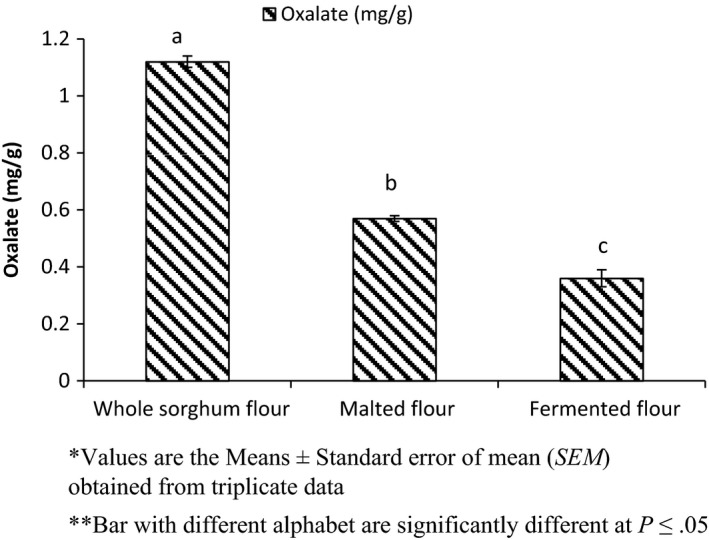
Effect of processing on oxalate of sorghum flour

The effect of processing on oxalate content is shown in Figure 4. The oxalate content of the unprocessed whole sorghum flour was found to be 1.12 mg/g, which reduced significantly during malting and fermentation. The oxalate content of germinated sorghum flour and fermented sorghum flour was 0.57 mg/g and 0.36 mg/g, respectively. The oxalate content of whole sorghum flour was similar to that reported by Opeyemi, Stephen, and Oluwatooyin (2016). Ojokoh (2014) revealed that oxalate content was reduced significantly during fermentation and malting.

#### 3.2.4 HCN (Hydrogen cyanide)

**Figure 5:**
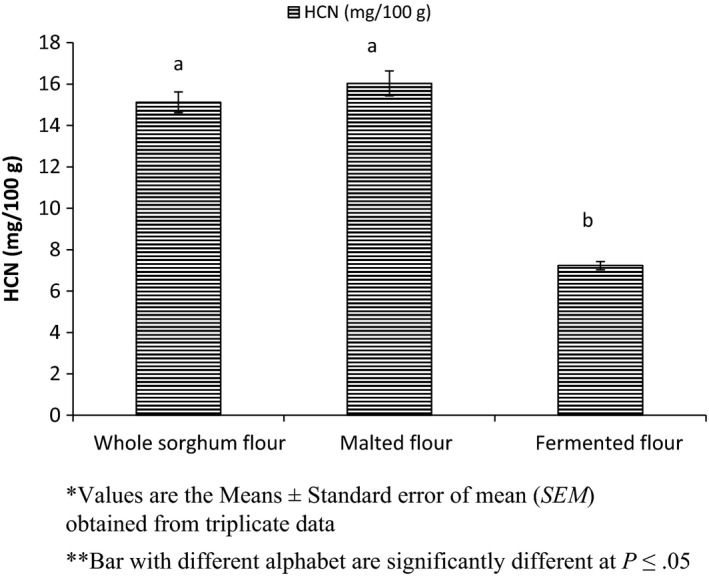
Effect of processing on hydrogen cyanide of sorghum flour

The effect of processing on hydrogen cyanide content is shown if Figure 5. The hydrogen cyanide (mg/100 g) of the whole sorghum flour, malted sorghum flour and fermented sorghum flour was found to be 15.16, 16.03 and 7.23, respectively. Malting process did not bring any significant change in HCN, but fermentation result in a significant decrease in HCN content.

Seeds may synthesized dhurrin during steeping and further during malting cyanogenic glycosides are biosynthesized from amino acid precursor such as tyrosine (Adewusi, 1983; Conn, 1980; Seigler, 1991). The health hazard is associated with incomplete removal of sprouts, as sprouts contain higher amount of hydrogen cyanide (Adewusi, 1983). Conn (1980) estimate that 50 mg of HCN is fatal to 70 kg man. Panasiuk and Bills (1984) revealed that nether neither drying at 50°C nor milling reduced the HCN content of sorghum. Decrease in HCN content in fermented sorghum flour might be due to microbial activity and pH drop during fermentation (Bhardwaj, Singh, Wangchu, & Sureja, 2005; Chima, Christian, Ekaette, & Ukpong, 2012; Ojokoh, 2014).

### 3.3 Functional characteristics of sorghum flour

#### 3.3.1 Bulk density and viscosity

**Figure 6:**
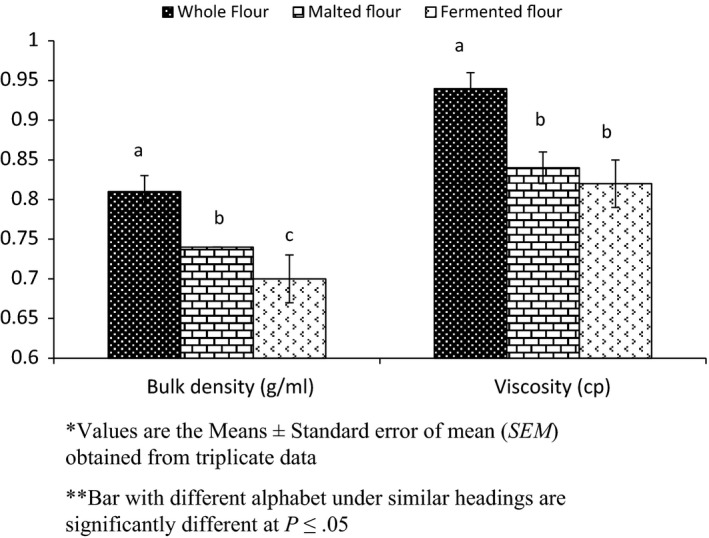
Effect of Processing on bulk density and viscosity of flour

The effect of processing on bulk density and viscosity of sorghum flouris shown in Figure 6. The bulk density of whole sorghum flour, malted flour and fermented flour were 0.81 g mL^−1^, 0.74 g mL^−1^ and 0.70 g mL^−1^, respectively. Elkhalifa, Schiffler, and Bernhardt (2005) also revealed reduction in bulk density of fermented sorghum flour. Break down of starch during fermentation reduce starch content and decrease the bulk density (Oti & Akobundu, 2008). Low bulk density flour was suitable for infant formulations (Nelson‐Quartey, Amagloh, Oduro, & Ellis, 2007). Low bulk weaning foods produced by germination and fermentation might be useful in many bakery products (Okoye, Ezigbo, & Animalu, 2010).

The viscosity from whole sorghum flour, malted flour and fermented flour were found to be 0.94cp, 0.84cp and 0.82cp, respectively. More soluble components were produced by the action of amylase and by the action of microbes during germination and fermentation, respectively. This may result in decrease in viscosity (Uvere, Ngoddy, & Nanyelugo, 2002). Reduction in carbohydrate and protein interaction changes in carbohydrate and protein interaction during treatment may produce a difference in viscosity (Odedeji & Oyeleke, 2011; Ohr, 2004).

#### 3.3.2 Oil absorption capacity and water absorption capacity

**Figure 7:**
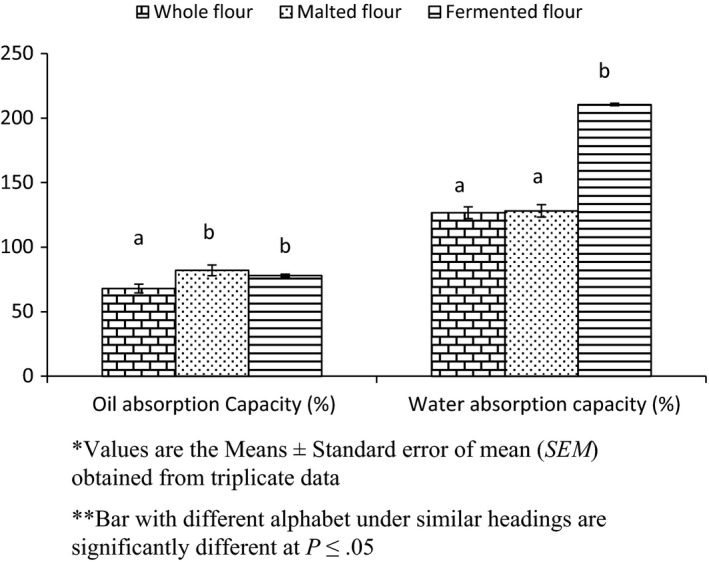
Effect of processing on water absorption and oil absorption of sorghum flour

The effect of processing on oil absorption capacity and water absorption capacity of sorghum flour is shown in Figure 7. Oil absorption capacity of the whole sorghum flour was found to be 68% and that of the germinated flour and fermented flour was found to be significantly increased to 82% and 78%, respectively. Fat absorption is mainly due to physical capturing and binding of fat to protein (Wang & Kinsella, 1976). It is an indication of the rate at which protein binds to fat in food formulations (Omimawo & Akubor, 2012). The increase in oil absorption capacity of the flour may help to maintain and improve mouth feel, if such flours are used as meat extenders etc. (Onuegbu, Nworah, Essien, Nwosu, & Ojukwu, 2013). Solubilization and dissociation of protein increase lipophilic constituent during germination and oil absorption capacity is increased (Deepali, Anubha, Preeti, & Krishi, 2013).

The water absorption capacity (WAC) of the Whole sorghum flour was found to be 126.72%. There is a significant increase in the water holding capacity of germinated and fermented sorghum flour. Water absorption of the flour is mainly depends upon protein, carbohydrate, their interaction and nature (Echendu, Onimawo, & Somtochi,^2004^; McWatters, Ouedraogo, Resurreccion, Hung, & Phillips, 2003; Onuegbu et al., 2013). There was no significant increase in WAC of malted flour, which might be due to less availability of polar amino acids in flours and due to lose association of amylose and amylopectin in the native granules of starch (Lorenz & Collins, 1990; McWatters et al., 2003). Traynham, Myers, Carriquiry, and Johnson(2007) revealed that WAC would affect the flour's thickness, viscosity, maintenance of freshness, and handling characteristics. The high water absorption capacity and its ability to increase water absorption when added to composite flour makes it a useful ingredient in food preparations such as soups, dairy products, beverages, coffee creamers, candies, gravies, and baked products (Sirivongpaisal, 2008).

## 4 CONCLUSION

Malting and fermentation significantly reduced antinutritional components of sorghum flour compared to malting and unprocessed. However, malting did not produce any significant changes in the hydrogen cyanide content of sorghum flour. Malting and fermentation reduce bulk density and viscosity but increased oil absorption capacity. Fermented sorghum flour was better due to reduce antinutritional factor and improved functional properties.

## CONFLICT OF INTEREST

None declared.
